# THz Shielding Properties of Optically Transparent PEDOT:PSS/AgNW Composite Films and Their Sandwich Structures

**DOI:** 10.3390/polym17030321

**Published:** 2025-01-24

**Authors:** Anton Voronin, Il’ya Bril’, Alexander Pavlikov, Mstislav Makeev, Pavel Mikhalev, Bogdan Parshin, Yuri Fadeev, Mikhail Khodzitsky, Mikhail Simunin, Stanislav Khartov

**Affiliations:** 1 Regional Educational and Scientific Center “Security” Bauman Moscow State Technical University, 105005 Moscow, Russia; pamikhalev@bmstu.ru (P.M.); parshbgal@bmstu.ru (B.P.); michanel@mail.ru (M.S.); 2 Federal Research Center «Krasnoyarsk Scientific Center», Siberian Branch, Russian Academy of Sciences (FRC KSC SB RAS), 660036 Krasnoyarsk, Russia; ellaijiah@gmail.com (I.B.); daf.hf@list.ru (Y.F.); stas_f1@list.ru (S.K.); 3 School of Non-Ferrous Metals and Material Science, Siberian Federal University, 660041 Krasnoyarsk, Russia; hahanka@yandex.ru; 4 LLC “Terahertz Photonics”, 191167 Saint-Petersburgh, Russia; khodzitskiy@yandex.ru

**Keywords:** silver nanowires, PEDOT:PSS, transparent electrode, electromagnetic shielding, THz time-domain spectroscopy

## Abstract

The modern pace of scientific and technological development dictates unprecedented requirements for the speed of information transfer. The THz range is considered one of the most promising and has been actively developing in recent years. Along with the need to develop transmitting devices, the demand for shielding materials in this range, including transparent ones, is also growing. In this work, we present two types of composite films based on silver nanowires and PEDOT:PSS. We characterized these composite films in terms of optoelectrical parameters, as well as shielding characteristics in the THz range. We found that our composite films have a sheet resistance (R□) of about 8.6 ± 1.2 Ω/□ with a transparency of about 83.41% and shielding efficiency is 25.85 dB in the THz region, which makes them excellent candidates for transparent shielding materials. We also made a bilayer sandwich structure from these composite films, which showed a shielding efficiency of about 49.34 dB in the range of 0.2–0.8 THz with a transparency of 66.33%. In addition, we assessed the possibility of real application of the structures in terms of stability to external conditions. Our composite films sustain atmospheric corrosion and maintain stable sheet resistance for 30 days.

## 1. Introduction

The discovery of radio waves triggered an unprecedented growth in the development of wireless communication systems. Wireless data transmission speeds doubling every 18 months [1]. The exponential trend of wireless capacity growth allows us to conclude that in 10 years, data transmission speeds of about tens of Gbit/s will be required [2]. Millimeter-wave communications (30–300 GHz) below 100 GHz have been officially adopted in recent 5G cellular systems. While the trend towards higher carrier frequencies is evident, millimeter-wave systems still struggle to support Tbit/s data rates as well as the aforementioned stringent quality of service requirements. Limited to a total sequential available bandwidth of less than 10 GHz in millimeter-wave systems below 100 GHz, spectrum efficiency must reach 100 bps/Hz, which is unprecedentedly difficult even using advanced physical layer techniques [3]. Electromagnetic waves in the frequency range of about 0.1–10 THz remain the least studied and developed across the spectrum, creating what is commonly known as the terahertz gap, mainly due to the lack of effective THz transceivers and antennas [4]. However, the aforementioned challenges have accelerated the filling of this gap over the past decade [5,6]. Together with the development of communication systems and electronic devices, flexible electronics [7] operating on THz waves, the problem of parasitic electromagnetic radiation and information security in this range will be relevant [8]. Current methods used at adjacent frequencies are either incompatible or inefficient at THz frequencies. For example, thermal detectors that convert incident radiation into heat are inefficient for THz frequencies because most wavelengths of THz radiation are not absorbed [9]. Thus, metal meshes cannot shield electromagnetic radiation with such a short wavelength because of the too-long period. Despite high electrical conductivity and optical transparency, doped oxides have serious drawbacks. They are usually expensive, fragile and difficult to integrate onto flexible substrates, which limits their application in optoelectronic devices [10]. Silver nanowires (AgNW) are an excellent alternative to transparent conducting oxides. The AgNW network combines optical transparency, high electrical conductivity, inexpensive fabrication and easy deposition on flexible substrates [11]. There are commonly used methods of depositing AgNW on a substrate: spray coating, spin-coating, and dip coating [12,13,14]. However, AgNW also have their own disadvantages. Due to their high specific surface area, they are susceptible to oxidation [15,16]. Strategies for increasing oxidation resistance include: AgNW coating with graphene and graphene oxide [1] PES coating [15,17], puttering of transparent conductive oxide (e.g., ITO) [16] and PEDOT:PSS [18]. The shielding properties of PEDOT:PSS [19,20] and PEDOT:PSS/AgNW [21] films have already been reported in the literature. The shielding of AgNW [22] in the terahertz range is also described. However, there are no papers on the shielding ability of PEDOT:PSS/AgNW films in the literature in the terahertz range. This inspired us to obtain PEDOT:PSS/AgNW sandwich films on a polymer substrate and to characterize their optical, electrical and shielding properties comprehensively.

## 2. Materials and Methods

### 2.1. AgNW Synthesis

High aspect ratio AgNW were synthesized by the solvothermal method: first, 0.3 g of polyvinylpyrrolidone (PVP) (M.W.—1.3 MDa)) (TW Reagents Panreac Castellar del Vallès, Barcelona, Spain) and AgNO_3_ (0.20 g, 1.18 mM) (EKOS, Moscow, Russia) were dissolved in 50 mL of ethylene glycol. A solution of FeCl_3_ (EKOS, Moscow, Russia) (200 μL, 12.5 μM) was then added and stirred at room temperature. The resulting mixture was then transferred to a 50 mL autoclave with a Teflon liner. This mixture of reagents was incubated at 130 °C for 8 h to complete the reaction. After synthesis, AgNW were subjected to a solvent exchange operation. For this purpose, the resulting solution in ethylene glycol was centrifuged at 10,000 rpm for 15 min using an OPN 16 laboratory centrifuge (Labtex, Moscow, Russia). After centrifugation, the supernatant was drained and the same amount of isopropyl alcohol was added. This operation was repeated 5 times until the complete removal of ethylene glycol, NO_3_^−^, Cl^−^ and Fe^3+^ ions and PVP residues.

### 2.2. Preparation of PEDOT:PSS/AgNW Composite Films

We chose two strategies for the formation of PEDOT:PSS/AgNW composite films. As a substrate, we used glass with a thickness of 1 mm and PET with a thickness of 300 μm

The first method is bilayer formation. In the beginning, AgNW film deposition using the spin-coating method. AgNW suspension in isopropanol with a concentration of ~0.5 mg/mL was used to apply the AgNW layer. The surface density of AgNW was determined by the number of application iterations; we used: 1, 3, 5, 7, 10, and 15 application iterations. The application speed was 2000 rpm, and the application time was 1 min. After application, the AgNW layer was dried in air at a temperature of 90 °C for 10 min. We then applied a PEDOT:PSS (Sigma-Aldrich, Saint-Louis, MO, USA) layer. For this application, we used a PEDOT:PSS suspension with a concentration of 5 mg/mL in a water/ethanol mixture. The PEDOT:PSS layer was applied at a deposition speed of 2000 rpm, the deposition time was 1 min. After application, the composite film was dried in air at a temperature of 120 °C for 10 min. We named these structures by the abbreviation P/AgNW.

The second method involves sequential application of PEDOT:PSS and AgNW layers. The formation of the composite film began and ended with PEDOT:PSS layers. The number of application iterations was the same as in the first method. That is—1, 3, 5, 7, 10, 15 iterations of application. For the application of PEDOT:PSS we used the same suspension in an ethanol–water mixture with a PEDOT:PSS concentration of ~0.5 mg/mL. One AgNW layer was encapsulated between the PEDOT:PSS layers. AgNW suspension in isopropanol with a concentration of ~0.5 mg/mL was used to form the AgNW layer. The application rate was 2000 rpm, and the application time was 1 min. After application, the samples were dried at a temperature of 120 °C for 10 min. We named these structures by the abbreviation P/AgNW/P.

### 2.3. Microscopy

The morphology and geometrical characteristics of a single AgNW were studied by transmission electron microscopy (TEM) on an HT7700 (Hitachi, Tokyo, Japan) at an accelerating voltage of 40–300 kV. The morphology of PEDOT:PSS/AgNW films was investigate using scanning electron microscopy (SEM) on a SU3500 microscope (Hitachi, Tokyo, Japan) at an accelerating voltage of 20 kV.

The surface morphology and thickness of the P/AgNW and P/AgNW/P composite films were studied by atomic force microscopy (AFM, Ntegra Prima (NT-MDT SI, Moscow, Russia). A cantilever with a curvature radius of 10 nm was used in the work.

### 2.4. Raman Spectroscopy

Raman spectra were measured using a 100× objective, 532 nm diode excitation laser. Exposure time was 15 s. and accumulated eight times. The Renishaw InVia Basis Raman (Renishaw, Wotton-under Edge, UK) spectrometer was used.

### 2.5. X-Ray Diffraction Analysis

X-ray diffractograms of the AgNW were taken using an X’Pert Pro MPD diffractometer (PANalytical, Lelyweg, The Netherlands) with a high-speed PIXcel detector in the angular range 30–90° 2Θ, with a step of 0.013°. The Ag lattice parameters were determined and refined using the full Rietveld approach by the difference derivative minimization (DDM) method.

### 2.6. Optoelectrical Properties

Optical Transmittance spectra of PEDOT:PSS/AgNW composite films were obtained in the range of 300–2600 nm (ultraviolet–visible–near-infrared UV–Vis–NIR) on a UV-3600i Plus spectrophotometer with an integrating sphere (Shimadzu, Kyoto, Japan). The scattering ability of AgNW films was evaluated by the Haze parameter. According to the standard D1003-13 “Standard Test Method for Haze and Luminous Transmittance of Transparent Plastics” [23], the Haze parameter is determined according to the following formula:

Haze,% = T_d_/T_t_, (1)

where T_d_ is diffuse Transmittance and T_t_ is total Transmittance. Measurements are carried out in two stages: in the first stage the Transmittance of the sample is measured directly—this is T_r_. In the second stage, the Transmittance in the sphere is measured—this is T_t_—then T_d_ = T_t_ − T_r_ is calculated. From the obtained data the Haze parameter is calculated.

The sheet resistance of PEDOT:PSS/AgNW composite films was measured by the four-probe method using a JG ST2258 four-point probe station (Suzhou Jingge Electronics Co., Suzhou, China) and a JG ST2558-F01 four-probe head (Suzhou Jingge Electronics Co., Suzhou, China).

### 2.7. THz Time-Domain Spectroscopy

Spectral properties of the P/AgNW and P/AgNW/P composite films on PET substrate in the terahertz frequency range were studied using the THz time-domain spectrometer. The spectrometer has a dynamic range > 70 dB at 0.4 THz, a spectral range till 1 THz. It provides real-time data acquisition with 10 spectra/s speed and spectral resolution up to 1 GHz. Photoconductive antennas were used as an emitter and a detector of THz radiation.

A composite films was placed in the focus area of the THz radiation between the parabolic mirrors of the THz-TDS spectrometer. The three THz waveforms were recorded for each sample: an empty space (air), a substrate and a sample on the substrate. Time averaging (over 100 spectra) was performed for each sample. The conversion of the signal from the time domain to the frequency domain was carried out using Fourier transformation. All measurements were carried out at a temperature of 20 ± 1 °C.

### 2.8. Environment Stability Tests

To test the atmospheric corrosion resistance of our composite films, we exposed AgNW, P/AgNW and P/AgNW/P composite film samples to air at 23 ± 1 °C for 30 days. All samples were obtained with 5 deposition iterations.

We measured the sheet resistance of our composite films for 30 days to test the resistance to atmospheric corrosion. Then, we plotted a graph of the sheet resistance versus time. SEM images were taken after 30 days.

To evaluate the mechanical resistance to cyclic bending load, we performed 1000 bending-unbending cycles with a radius of 5 mm using a self-made bending template.

## 3. Results

### 3.1. AgNW and PEDOT:PSS Characterization

First, we characterized the AgNW that we synthesized. To evaluate their properties, we used TEM, SAED, UV–Vis spectroscopy and Raman spectroscopy.

**Figure 1:**
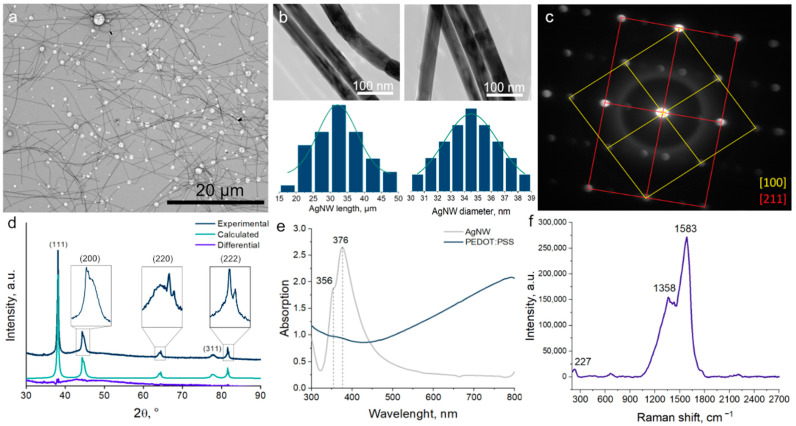
TEM image of AgNW (**a**); HRTEM image of AgNW, AgNW length and AgNW diameter histograms (**b**); selective area electron diffraction (SAED) (**c**); X-ray diffraction (XRD) (**d**); UV–Vis absorption spectra of AgNW and PEDOT:PSS (**e**); Raman spectra of AgNW (**f**).

Figure 1a,b shows TEM images of the AgNW. We made histograms of the length and diameter distributions using these images. Both parameters were fit to the Gaussian curve. The calculated average length of the AgNW is 32 ± 13 µm. The calculated average diameter of the AgNW is 36 ± 5 nm. With these dimensions, an aspect ratio of about 888:1 was obtained, which is quite close to the 1000:1 that is accepted in the literature as a high one [24].

Figure 1c shows the SAED pattern. We see that AgNW have a face-centric tetragonally distorted lattice. We also observe five-fold twinning in AgNW. AgNW consist of five tetrahedra, with a small correspondence angle between them [25]. Figure 1d shows the XRD of AgNW refined by the Rietveld method. We see the c peaks of crystalline silver at 2θ = 38.13°, 44.4°, 64.4°, 77.47° and 81.5° were indexed to the (111), (200), (220), (311) and (222) reflections of metallic Ag [26]. Our AgNW consist of two phases. First phase has a cubic lattice Fm3m and parameters a = b = c = 4.0868(7) Å. The second phase has a tetragonally distorted F4/mmm lattice and parameters a = b = 4.058 Å (3), c = 4.164 Å (8). AgNW have an orientation along the (011) plane [27].

In Figure 1e we see the UV–Vis spectra of AgNW and PEDOT:PSS. AgNW have absorption peaks at 356 nm and 376 nm. These peaks are related to the phenomenon of plasmon resonance, and their position agrees with the available literature data [28,29]. According to the data presented in [30], the absorption peaks for AgNW with diameters of 36 ± 5 nm should be in the UV region. The slight broadening of the peak is due to the same small spread of AgNW diameters. PEDOT:PSS absorption spectrum shows an increasing trend after moving from UV to VIS region. This trend agrees with the literature results [31].

Figure 1f shows the Raman spectrum of the AgNW that we synthesized. We see three distinct peaks at 227, 1358, and 1583 cm^−1^, respectively. According to the literature, the peak at 227 cm^−1^ is associated with the stretching vibrations of the Ag-O bond. The peak at 1583 cm^−1^ is attributed to the stretching oscillation of the C=O bond. Both of these peaks confirm the presence of a PVP layer on the surface of the AgNW [32]. After characterizing the materials individually, we proceeded to create composite films based on AgNW/PEDOT:PSS.

### 3.2. PEDOT:PSS/AgNW Composite Films Preparation and Characterization

**Figure 2:**
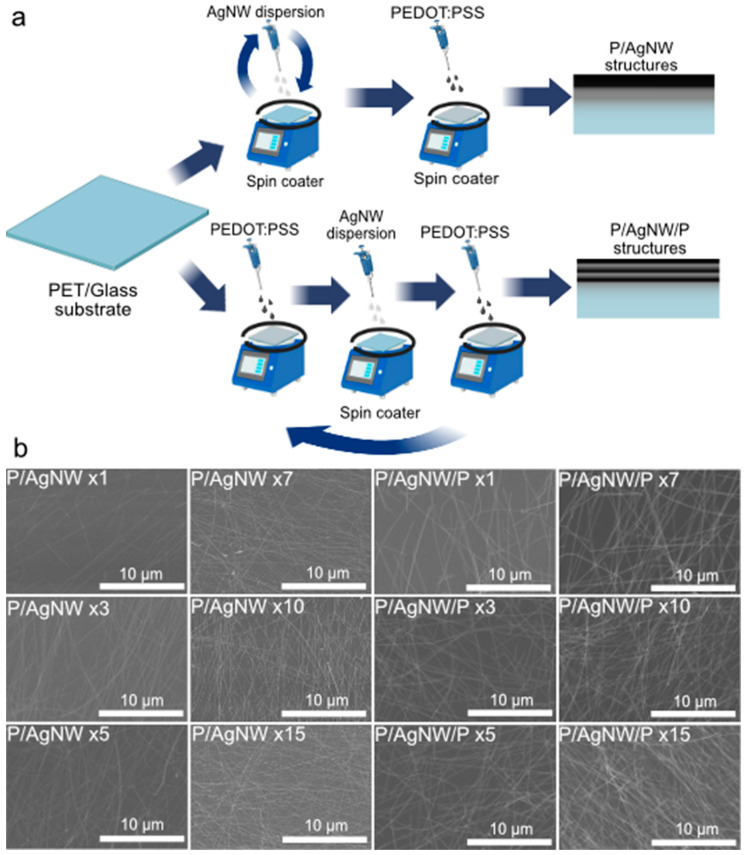
Composite films preparation process illustration (**a**); SEM-images of P/AgNW and P/AgNW/P composite films (**b**).

We used the spin-coating technique to create PEDOT:PSS/AgNW composite films. Spin-coating is popular a technology used to create uniform thin films with a thickness of micrometers and nanometers [33]. We have two options for composite film formation. The first is to deposited AgNW, then deposited PEDOT:PSS. This will result in a bilayer structure, which we named by the abbreviation P/AgNW. The second method is layer-by-layer alternation of AgNW and PEDOT:PSS. As a result, we will obtain a multilayer composite film, which we named by the abbreviation P/AgNW/P. A schematic representation of the sample preparation is shown in Figure 2a.

P/AgNW samples were prepared by this method: AgNW were deposited on the substrate at 0.1 mL in one or several deposition iterations (3, 5, 7, 10, or 15). PEDOT:PSS was then deposited on top of the AgNW film in the same way.

The P/AgNW/P samples were prepared by this method: AgNW and PEDOT:PSS layers were deposited one by one, starting and ending with a layer of PEDOT:PSS. The number of iterations was 1, 3, 5, 7, 10, or 15 as in the case of P/AgNW. The number of deposition iterations was changed to vary the surface density of the AgNW film.

We obtain SEM images of P/AgNW and P/AgNW/P composite films with different iteration numbers during preparation. The results are shown in Figure 2b. The AgNW surface density increases with the iteration number for both composite film types. However, in the case of P/AgNW/P, we see that the composite films have higher contrast. This can be attributed to the fact that the P/AgNW samples are coated with a thick layer of PEDOT:PSS on top.

**Figure 3:**
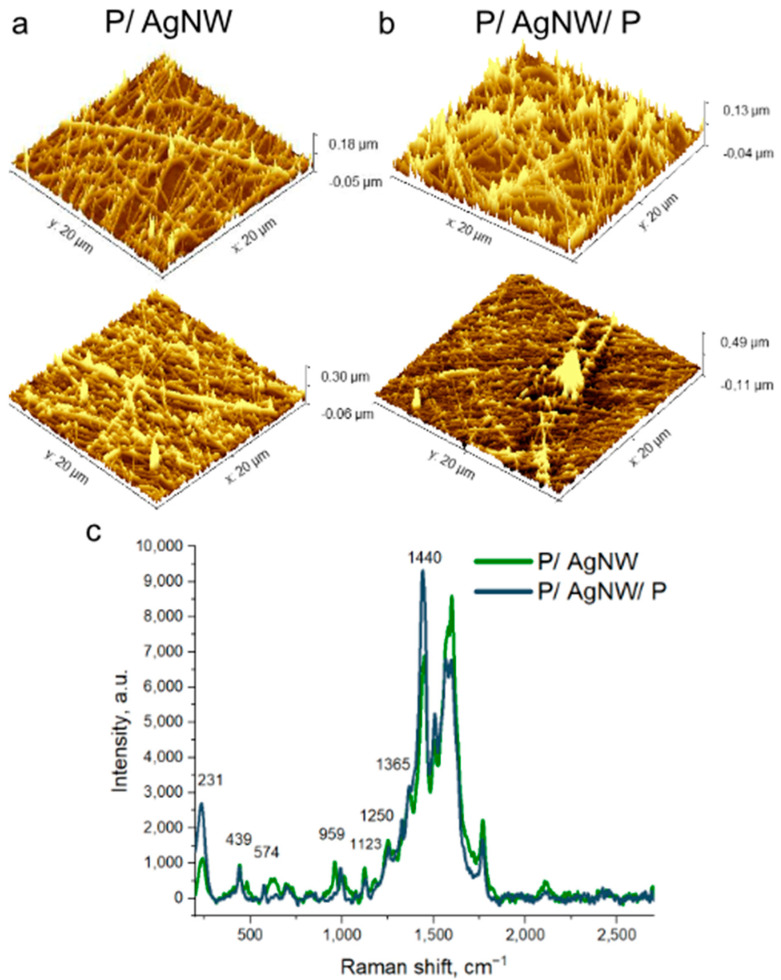
AFM-images of P/AgNW composite films obtained by 5 deposition iterations (**top picture**) and 15 deposition iterations (**bottom picture**) (**a**); P/AgNW/P composite films obtained by 5 deposition iterations (**top pictures**) and 15 deposition iterations (**bottom pictures**) (**b**); Raman spectra of P/AgNW and P/AgNW/P films (**c**).

Next, we investigate the local morphology and thickness of the composite films as a function of the number of iterations using atomic force microscopy (AFM). Figure 3 shows the AFM results for P/AgNW (Figure 3a) and P/AgNW/P (Figure 3b) composite films with the same number of deposition iterations (5 and 15 iterations, respectively). The full AFM data on the morphology of the two types of composite films are given in the Supplementary Materials (Table S1). The average thickness of the two types of composite films differs slightly. This suggests that increasing the number of deposition iterations leads to a higher surface-filling factor of composite films. However, the increase in thickness is much less pronounced. The following conclusions can be drawn from the study: the thickness of the composite films is extremely uneven, which significantly complicates the analysis of their thickness. In some regions of the film, the thickness may be only a few nanometers, while in others, where several AgNW intersect, the thickness of which in our case can be 3–4 diameters of one AgNW, i.e., 100–130 nm.

In Figure 3c, we see the Raman spectra of P/AgNW and P/AgNW/P composite films obtained using a 785 nm laser wavelength. We see the peaks of the main oscillatory modes of PEDOT at wavelengths of 231, 439, 574, 959, 1123, 1250, 1365, and 1440 cm^−1^, respectively [34]. For example, the main peak at 1440 cm^−1^ is related to the symmetric stretching of the aromatic bond C=C. We also see a peak at 1365 cm^−1^ which is related to the asymmetric stretching of C_β_–C_β_ and a peak at 1250 cm^−1^ related to the intra-ring stretching of C_α_–C_α_ [35]. The peak at 1578 cm^−1^ is due to the stretching of the C=O bond, which is present in both PEDOT:PSS and PVP molecules. The peak at 1583 cm^−1^ belongs to AgNW, as mentioned earlier. All the above peaks have the same position on both P/AgNW and P/AgNW/P composite films.

### 3.3. Optoelectrical Properties

Achieving an optimal balance between optical and electrical properties is crucial in the development of transparent shielding materials [36]. To characterize the optical properties of P/AgNW and P/AgNW/P composite films, we measured their Transmittance and Haze in the UV–Vis–NIR range.

**Figure 4:**
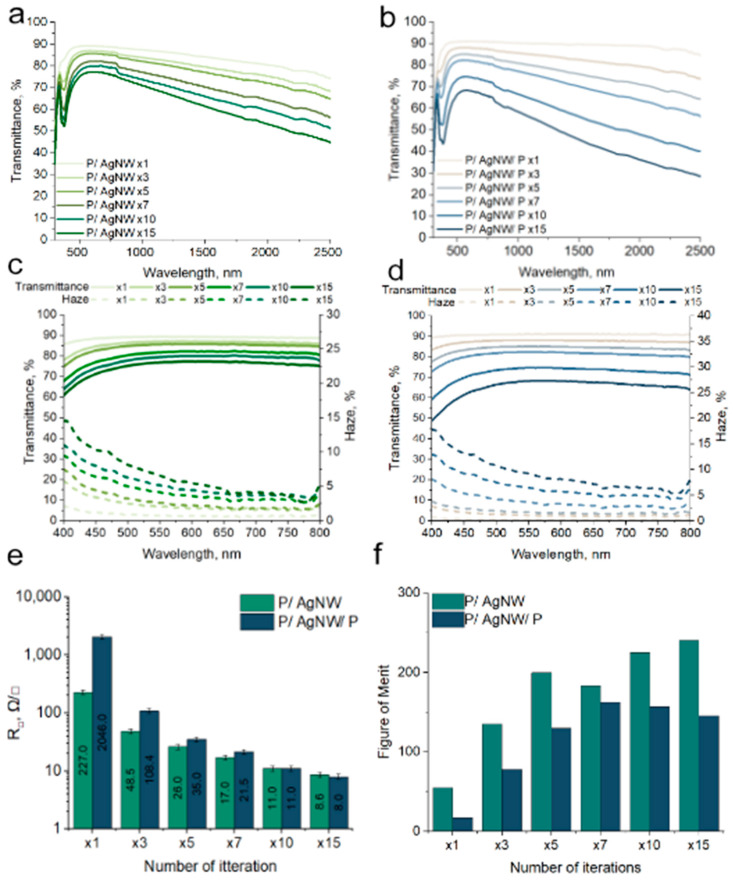
Optical Transmittance in UV–Vis–NIR range of P/AgNW composite films (**a**) and P/AgNW/P composite films (**b**); Transmittance spectra and Haze in the visible range of P/AgNW (**c**) and P/AgNW/P composite films (**d**); sheet resistance of P/AgNW and P/AgNW/P composite films (**e**); figure of merit (FoM) of P/AgNW and P/AgNW/P composite films (**f**).

The results of the Transmittance measurements in the range of 300–2600 nm are shown in Figure 4a for P/AgNW composite films and in Figure 4b for P/AgNW/P composite films on glass substrates. Both types of composite films exhibit a minimum Transmittance at 376 nm, which can be explained by the plasmon resonance phenomenon associated with AgNWs [37].

A uniform decrease in Transmittance is observed in the IR range of 780-2600 nm. The slope of the transmission spectra is related to the electronic properties of PEDOT:PSS. A broad optical absorption band, which begins in the IR range, arises as a result of electron transitions from the valence band to the polariton or bipolaron band. This absorption band then merges with transitions from the valence band to the conduction band [38].

Figure 4c,d shows the optical Transmittance and Haze in the visible range for the P/AgNW and P/AgNW/P composite films. The Haze value shows the ratio of diffuse light Transmittance to total light Transmittance. Since our P/AgNW and P/AgNW/P composite films represent a network of randomly arranged AgNW covered by PEDOT:PSS, they scatter incident radiation [39]. Haze spectra tend to increase with decreasing wavelength. Increasing the density of AgNW in the film leads to an increase in Haze. For example, for P/AgNW films, Haze increases from 0.71% (×1) to 6.54% (×15) at a wavelength of 550 nm. For P/AgNW/P films, Haze increases from 0.31% (×1) to 9.22% (×15) at a wavelength of 550 nm. Haze has a critical dependence on the AgNW diameter [40] and their surface density. The difference in the Transmittance and Haze between the P/AgNW and P/AgNW/P composite films is due to the peculiarities of the film formation methods. As we have already mentioned earlier, P/AgNW composite films are created by layer-by-layer deposition of AgNW and then deposition of PEDOT:PSS on top of these AgNW. With this method, the effect of saturation of the AgNW surface density occurs. In this method, the excess AgNW will be swept away from the substrate surface during spin-coating. The effect of saturation of the surface filling factor is observed for P/AgNW composite films, the Transmittance and Haze of the composite films change disproportionately to the number of deposition iterations.

The opposite situation is observed for P/AgNW/P composite films. In their case, Transmittance and Haze change proportionally with the increase in the number of iterations. This is due to the fact that the method for creating P/AgNW/P composite films involves layer-by-layer deposition of AgNW and PEDOT:PSS. In this case, PEDOT:PSS becomes a binder for each of the AgNW layers in the P/AgNW/P composite film. This allows for the deposition of a larger amount of AgNW. This explains the difference in Transmittance and Haze values for P/AgNW and P/AgNW/P composite films.

Sheet resistance is an important parameter for the design and selection of materials for EMI compatibility applications. In Figure 4e, we see the sheet resistance values obtained for P/AgNW and P/AgNW/P composite films. The first thing we see is a trend towards decreasing sheet resistance with an increasing number of deposition iterations. This is due to the increase in the amount of AgNW in the film and the number of electrical contacts between them. For example, we see that the sheet resistance drops from 227 Ω/□ for 1 iteration to 8.6 Ω/□ for 15 iterations for P/AgNW composite films. Another one example: for P/AgNW/P from 2046 Ω/□ for 1 iteration to 8 Ω/□ for 15 iterations. At 10 and 15 iterations we observe a slight decrease in resistance, which may indicate reaching a plateau with a further increase in the number of iterations of layer application. Thus, we can conclude that the most optimal number of iterations is 10–15. And with further increase number of iterations, the resistance will change insignificantly. It is also important to evaluate the applicability of the formation method for different types of substrates. The differences in sheet resistance values for P/AgNW and P/AgNW/P composite films at small quantities of deposition iterations are due to the specifics of the method. When creating P/AgNW composite films, we first deposit AgNW layers and then coat them with PEDOT:PSS. In the case of P/AgNW/P composite films, we deposit AgNW and PEDOT:PSS layer by layer. With this method, the quality of contacts between AgNW will be worse than in the case of P/AgNW. A worse quality of contacts between AgNW will have a greater effect on sheet resistance at small quantities deposition iterations, which is what we observe in Figure 4e. We present data on the sheet resistance of P/AgNW and P/AgNW/P composite films on a PET substrate (Figure S1). The figure shows that the composite film formation method is universal for all types of substrates that we used. The exception is composite films obtained by one deposition iteration.

Evaluating the relation between the values of sheet resistance and Transmittance in the visible range is useful for transparent shielding materials. The relation between these two values is characterized by the figure of merit (FoM) parameter. It can be calculated using the equation

(2) $$FoM=\frac{Z_{0}}{2R\left(\frac{1}{\sqrt{T}}-1\right)},$$

where is *T* is the optical Transmittance at a wavelength of 550 nm; sheet resistance is the sheet resistance; Z_0_ = 377 Ω is the impedance of free space.

We operate with the Transmittance of the composite film and not taking into account the substrate, which reduces the Transmittance in the visible range by 8–10% as a rule. To compensate for the substrate in the first approximation of a flat spectrum we use the equation

(3) $$T_{f}=\frac{T_{f+s}}{T_{s}},$$

where *T_f_* is the Transmittance of the film under study, *T_s_* is the Transmittance of the substrate, and *T_f+s_* is the total Transmittance of the structure under study. The Transmittance spectrum of the glass on which we studied the optoelectrical properties is shown in Figure S3. The Transmittance of the glass substrate at a wavelength of 550 nm was 92%.

We calculated FoM for P/AgNW and P/AgNW/P composite films. The results are shown in the histograms in Figure 4f. We see a trend of increasing FoM of P/AgNW composite films with increasing of deposition iterations. For P/AgNW/P composite films, we see a peak at 7 iterations, and then a trend of decreasing FoM for 10 and 15 iterations. The difference in FoM trends for the two different composite films is explained by their Transmittance. Composite films have close sheet resistance values, but the drop in Transmittance for P/AgNW/P composite films is higher than for P/AgNW. The maximum FoM value for P/AgNW = 231.13, and for P/AgNW/P = 159.18.

Complete data on the optoelectrical parameters of P/AgNW and P/AgNW/P composite films are given in Table 1.

### 3.4. THz Shielding Properties of P/AgNW and P/AgNW/P Composite Films

Shielding is the weakening of electromagnetic radiation. This definition is valid both in the case of the radio frequency range [41] and the terahertz range [42]. The shielding efficiency (SE) is related to the Transmittance (*T*) in the THz range by the equation

(4) SE=−10×lg⁡T

The Transmittance provides information to characterize the shielding properties of the PEDOT:PSS/AgNW composite films obtained by us.

**Figure 5:**
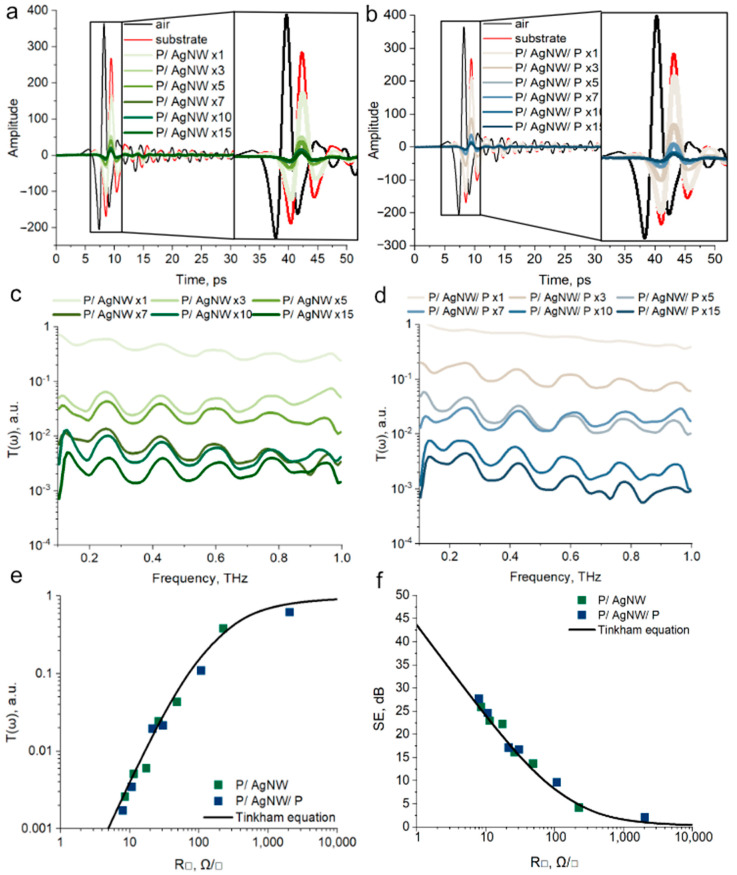
Time waveforms of THz pulses for P/AgNW (**a**) and P/AgNW/P composite films (**b**); Transmittance spectra in the range of 0.1–1 THz for P/AgNW (**c**) and P/AgNW/P composite films (**d**); dependences of average Transmittance on the sheet resistance value (**e**); dependences of SE on the sheet resistance value (**f**).

The time waveforms of THz pulses passed through PEDOT:PSS/AgNW composite films on PET substrates are shown in Figure 5. The P/AgNW composite films are shown in Figure 5a, and the P/AgNW/P composite films are shown in Figure 5b.

Each graph also includes the amplitudes of pulses that passed through free space and a PET substrate for normalization. The data indicate that the PET substrate contributes to a reduction in the amplitude of the incident THz pulses, with an accompanying phase shift due to retardation effects linked to PET polarization. A further decrease in pulse amplitude is observed after the deposition of PEDOT:PSS/AgNW composite films onto the PET substrate. The decrease in the amplitude of THz pulses is proportional to the decrease in the sheet resistance of composite films.

The Transmittance spectra of P/AgNW and P/AgNW/P composite films, measured in the 0.1–1 THz range, are presented in Figure 5c and Figure 5d, respectively. All spectra are normalized to the Transmittance of the substrate, allowing us to determine the Transmittance characteristics of our composite films. The Transmittance of the samples decreases as the AgNW surface density increases, which is with the sheet resistance trend. This trend is observed for both the P/AgNW (Figure 5c) and P/AgNW/P (Figure 5d) series. The spectra exhibit a relatively flat profile with low-amplitude oscillations. Therefore, the variations in sheet resistance are agreed with the average Transmittance values across the 0.1–1 THz range. Given the uniformity of the spectra over this frequency range, the composite films can be effectively described by their average Transmittance.

The Transmittance of thin films in the THz range can be calculated using the Tinkham equation [43].

(5) Tω=(EAgNW−PEDOT:PSS(ω)EPET(ω))2=(1+n1+n+Z0σ(ω)d)2 =(1+n1+n+Z0Rs)2,

where E_PET_ (ω) and E_AgNW-PEDOT:PSS_ (ω) are the amplitudes of the electric field of the THz wave transmitted through the substrate and the substrate with the PEDOT:PSS/AgNW composite film; σ(ω) is the frequency dependence of the conductivity of the PEDOT:PSS/AgNW composite film, *d* is the thickness of the PEDOT:PSS/AgNW composite film, *Z_0_* is the impedance of free space equal to 377 Ω, and n = 1.57 is the refractive index of the PET substrate. Based on the considerations that in the THz range the frequency-dependent conductivity can be replaced by a static value and the following replacement is accepted, *R_s_* = 1/σ(ω)*d*. Based on these assumptions, we can obtain the calculated dependence of the transmittance in the THz range on the sheet resistance value, according to the Tinkham Equation (5), which can be compared with the experimental values of the transmittance for the P/AgNW and P/AgNW/P composite films. Figure 5e shows the averaged experimental values of the transmittance in the range of 0.1–1 THz for the P/AgNW and P/AgNW/P composite films and a comparison with the dependence obtained using the Tinkham equation. We see good agreement between experimental and calculated data.

The transmittance values alone cannot provide sufficient insight. Therefore, it is more informative to convert the data into SE values. Figure 5f shows the averaged experimental SE values as well as the values calculated using Equation (4). The graph demonstrates excellent agreement between the experimental data and the values predicted by the Tinkham equation. The maximum average SE value observed experimentally was 25.86 dB for the P/AgNW composite film (15 deposition iterations) and 27.66 dB for the P/AgNW/P composite film (15 deposition iterations), respectively.

To highlight our results, we compared specific shielding efficiency (SSE_T_) with other materials. SSE_T_ is a parameter that takes into account the density of the material and its thickness. It is calculated using the following formula:

(6) $$SSE_{T}=SE/\rho \times t,$$

where ρ—density in g/cm^3^ and *t*—thickness in mm [44].

**Figure 6:**
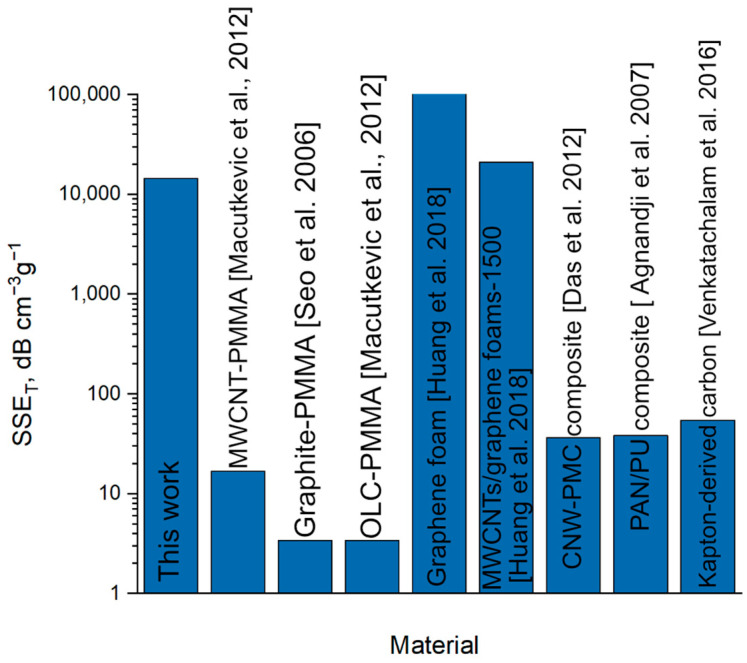
SSE_T_ comparison between our composite films and other materials [45,46,47,48,49,50,51].

Figure 6 compares the (SSE_T_) of our material with that of other materials, including MXenes, graphene, carbon nanotube composites, and others. As shown, our results are not surpassed only by graphene foam-based materials, which have extremely low sheet resistance and density. However, these materials are inferior to our composite films in terms of mechanical properties and transparency.

The achievement of such high results by our composite films is explained by several factors: First, low density, due to the method of obtaining our films. Second, low film thickness not exceeding 100 nm (Table S1). Third, high shielding efficiency.

### 3.5. THz Shielding Properties of Bi- and Trilayer Sandwich Structures Based on P/AgNW Composite Films

The assembly of sandwich structures is a highly effective method for enhancing shielding properties of materials [52]. In the radio frequency range of 1–18 GHz, two mesh layers with low sheet resistance are typically sufficient [53,54,55,56,57]. However, there are also instances where materials with three or more functional layers have been employed to further improve performance [58,59].

We made two types of sandwich structures—bilayer and trilayer—to achieve materials with high shielding effectiveness. Based on optoelectrical properties (Figure 4), we selected P/AgNW composite films to form these sandwich structures, as they exhibited a higher FoM. The sandwich structures consisted of the following composite film configurations: bilayer P/AgNW (×10)/PET/P/AgNW (×15) and trilayer P/AgNW (×7)/PET/P/AgNW (×10)/PET/P/AgNW (×15). The P/AgNW composite films were assembled such that a PET spacer was formed between the conducting layers.

**Figure 7:**
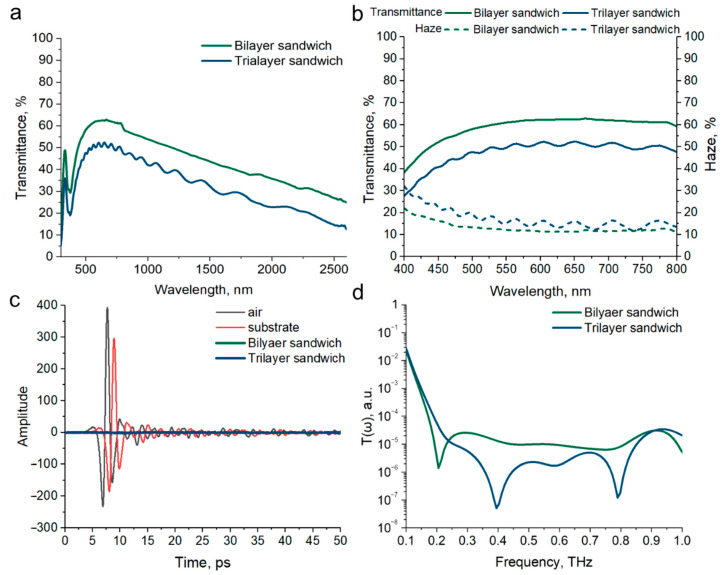
Transmittance bi- and trilayer sandwich structures based on P/AgNW composite films in the range of 300–2600 nm (**a**); optical Transmittance and Haze spectra bi- and trilayer sandwich structures based on P/AgNW composite films in the visible range (**b**); time shapes of THz pulses transmitted through bi and trilayer sandwich structures (**c**); Transmittance spectra bi- and trilayer sandwich structures in the range of 0.1–1 THz (**d**).

We evaluate the optical properties of the resulting sandwich structures. The Transmittance in the 300–2600 nm range for both bilayer and trilayer sandwich structures is shown in Figure 7a. The spectral shape of the bilayer and trilayer structures closely resembles that of single-layer composite films, and the Transmittance decreases as the number of composite films in the sandwich structure increases, proportional to the transmittance of each composite film. Figure 7b shows the Transmittance and Haze in the visible range. For the bilayer sandwich structures, the absolute transmittance at 550 nm is 61.03%, and the transmittance without taking into account the contribution of the substrate, according to Equation (3), is 66.33%. For the trilayer sandwich structures, the absolute transmittance at 550 nm is 49.55%, and the transmittance without taking into account the contribution of the substrate is 53.83%.

The frequency dependence of Haze increases with decreasing wavelength, according to Rayleigh’s law, as in the case of single-layer composite films. It is also clearly seen that the absolute value of Haze is additively composed of the Haze values of individual composite films. At a wavelength of 550 nm, the Haze parameter value is 12.25% for a bilayer sandwich structure and 15.21% for a trilayer sandwich structure, respectively.

The time waveforms of THz pulses passed through bi- and trilayer sandwich structures are shown in Figure 7c. The observed THz pulse amplitude after passing through a bi- and trilayer sandwich structures is significantly lower than in the case of single-layer composite films. This indicates a significant increase in the shielding properties.

The Transmittance spectra of bi- and trilayer sandwich structures in the 0.1–1 THz range are shown in Figure 7d. The spectra show one peak for the bilayer sandwich structure and two peaks for the trilayer sandwich structure. The average shielding efficiency in the 0.2–0.8 THz range is 49.34 dB for the bilayer sandwich structure and 56.66 dB for the trilayer sandwich structure. These results demonstrate high shielding efficiency over a broad frequency range. These results indicate that the multilayer configuration is highly effective for THz shielding applications. It is important to note that, in future studies, varying the spacer thickness should also be considered, as this parameter has been shown to be an effective means of further enhancing the shielding efficiency value [60].

### 3.6. Environment Stability of P/AgNW and P/AgNW/P Composite Films

Metals are trends to corrosion under atmosphere conditions. In the case of silver, atmospheric corrosion typically leads to sulfidation rather than the formation of silver oxide [61]. This process was named tarnishing, The corrosion of silver-based nanostructures has been well studied, and it usually leads to the formation of a silver sulfide nanocrystal shell. This process limits the applicability of AgNW as transparent shielding materials, as both corrosion and the resulting degradation of their electrical properties hinder their performance.

One way to protect AgNW from atmospheric corrosion is to coat them with a polymer. For example, cover it with polymer [62]. In this case, the polymer will act as an insulating layer, protecting the AgNW from the influence of air. For our applications, it is critical to maintain the electrical conductivity of the structure. This is what motivated us to integrate AgNWs into the conducting polymer PEDOT:PSS.

**Figure 8:**
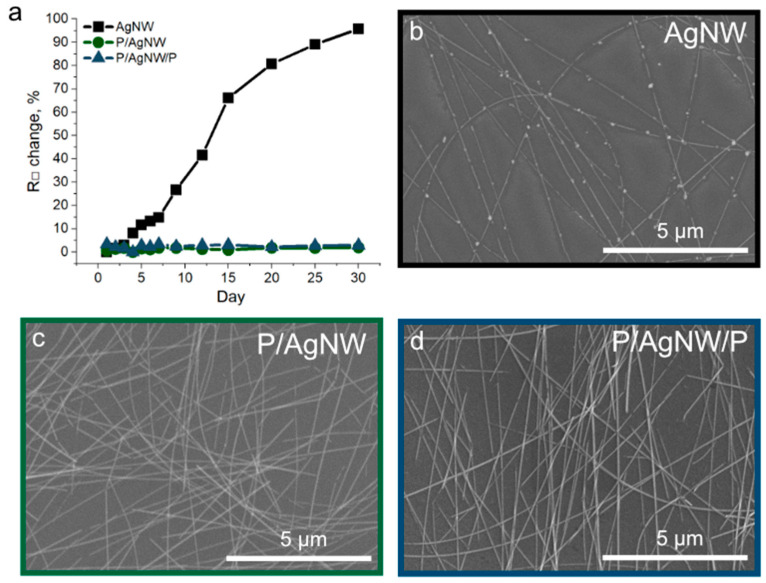
Stability of sheet resistance of AgNW films, P/AgNW, and P/AgNW/P composite films over time under the influence of the environment (**a**); SEM images of AgNW films (**b**); P/AgNW composite film (**c**); P/AgNW/P composite film (**d**).

We tested the stability of our composite films to atmospheric conditions for a month. The results are shown in Figure 8.

Figure 8a shows the results of observing the change in sheet resistance over time under the influence of ambient air. As we can see, the AgNW films were subjected to the atmospheric corrosion process, as a result of which their resistance increased from 13.45 Ω/□ to 26.33 Ω/□ (at 95,76%) in 30 days. The fact of atmospheric corrosion is also confirmed by the SEM image Figure 8b.

At the same time, the P/AgNW and P/AgNW/P composite films were not subjected to corrosion, and their sheet resistance remained the same (there is a scatter associated with the measurement error). The absence of the atmospheric corrosion process is also confirmed by the SEM image of P/AgNW (Figure 8c) and P/AgNW/P (Figure 8d) composite films.

In addition, we conducted a cyclic test for 1000 bending and unbending cycles. The test results are shown in Figure S4 in Supplementary Materials.

## 4. Conclusions

In this study, we investigated the properties of PEDOT:PSS/AgNW composite films deposited on glass and PET substrates. Each films component was characterized individually: AgNW was analyzed using TEM, SAED, UV–Vis spectroscopy, and Raman spectroscopy.

We proposed and evaluated two methods for forming composite films: P/AgNW and P/AgNW/P. Both types of composite films were characterized in terms of their optical and electrical properties. The P/AgNW method shows better optical and electrical properties. For example, P/AgNW films reach the Transmittance at about 83.41%, compared to 74.17% for P/AgNW/P films. A similar trend was observed for Haze, where P/AgNW films made by 15 iterations have a Haze of about 6.54% at 550 nm, compared to 9.22% for P/AgNW/P films.

P/Ag NW composite films are characterized by an average shielding efficiency of 25.86 dB in the range of 0.1–1 THz with 15 deposition iterations. P/Ag NW/P composite films have an average shielding efficiency of 27.66 dB in the range of 0.1–1 THz with 15 deposition iterations.

In addition to the shielding properties of monolayer films, we also evaluate the optical and shielding properties of sandwich structures based on our composite films. The bilayer sandwich structure consisting of P/AgNW (×10)/PET/P/AgNW (×15) has a transmittance of about 66.33% at 550 nm with an average shielding efficiency of 49.34 dB in the range of 0.2-0.8 THz. The trilayer sandwich structure consisting of P/AgNW (×7)/PET/P/AgNW (×10)/PET/P/AgNW (×15) has a transmittance of about 53.83% at 550 nm with an average shielding efficiency of 56.66 dB in the range of 0.2-0.8 THz.

These results highlight that the composite film-obtaining methods described in this study enable the development of durable, transparent shielding materials suitable for applications in the THz range.

## Figures and Tables

**Table 1:** Optoelectrical parameters of P/AgNW and P/AgNW/P composite films on a glass substrate.

Films Type	R, Ω/□	*T_f-s_* (550 nm), %	*T_f_* (550 nm), %	*Haze* (550 nm), %	*FoM*
P/AgNW (×1)	227 ± 34	89.22	96.97	0.71	53.66
P/AgNW (×3)	48.5 ± 7.2	86.89	94.44	2.01	134.05
P/AgNW (×5)	26 ± 4	85.56	93.1	2.58	197.23
P/AgNW (×7)	17.0 ± 2.6	81.69	88.79	4.13	175.45
P/AgNW (×10)	11.0 ± 1.7	79.38	86.28	5.16	214.98
P/AgNW (×15)	8.6 ± 1.2	76.74	83.41	6.54	231.13
P/AgNW/P (×1)	2046 ± 307	91.01	98.92	0.31	16.92
P/AgNW/P (×3)	108.4 ± 16.2	88.14	95.81	1.11	80.38
P/AgNW/P (×5)	35.0 ± 5.2	85.06	92.45	1.71	134.45
P/AgNW/P (×7)	21.5 ± 3.2	82.31	89.46	3.68	152.87
P/AgNW/P (×10)	11.0 ± 1.6	74.63	81.11	6.47	159.18
P/AgNW/P (×15)	8.0 ± 1.2	68.24	74.17	9.22	146.03

## Data Availability

Data are contained within the article or Supplementary Materials.

## Supplementary Materials

The following supporting information can be downloaded at: https://www.mdpi.com/article/10.3390/polym17030321/s1, Figure S1: Sheet resistance of P/AgNW and P/AgNW/P composite films on PET substrate; Figure S2: Homogenity of P/AgNW composite film in 10 iterations; Figure S3: Glass transmittance spectra; Figure S4: Stability to bending of P/AgNW (7 it.) structure; Table S1: Morphological characteristics of P/AgNW and P/AgNW/P structures.

## References

[1] Cherry S. (2004). Edholm’s law of bandwidth. IEEE Spectr..

[2] Kleine-Ostmann T., Nagatsuma T. (2011). A Review on Terahertz Communications Research. J. Infrared Millim. Terahertz Waves.

[3] Akyildiz I.F., Han C., Hu Z., Nie S., Jornet J.M. (2022). Terahertz Band Communication: An Old Problem Revisited and Research Directions for the Next Decade. IEEE Trans.Commun..

[4] Liu L., Das A., Megaridis C.M. (2014). Terahertz Shielding of Carbon Nanomaterials and Their Composites—A Review and Applications. Carbon.

[5] Song H.-J., Lee N. (2021). Terahertz Communications: Challenges in the Next Decade. IEEE Trans. Terahertz Sci.Technol..

[6] TERAHERTZ RF ELECTRONICS AND SYSTEM INTEGRATION. https://ieeexplore.ieee.org/document/7931729.

[7] Wen J., Zhou L., Ye T. (2024). Polymer ionogels and their application in flexible ionic devices. SmartMat.

[8] He Z., Liu Z., Liu B., Wang K., Dong X., Zhang Z., Chen C., Wang M., Liu J., Huang W. (2024). Thermally-induced phase fusion and color switching in ionogels for multilevel information encryption. Chem. Eng. J..

[9] Venkatachalam S., Zeranska-Chudek K., Zdrojek M., Hourlier D. (2020). Carbon-Based Terahertz Absorbers: Materials, Applications, and Perspectives. Nano Sel..

[10] van Hoof N., Parente M., Baldi A., Rivas J.G. (2020). Terahertz Time-Domain Spectroscopy and Near-Field Microscopy of Transparent Silver Nanowire Networks. Adv. Opt. Mater..

[11] De S., Higgins T.M., Lyons P.E., Doherty E.M., Nirmalraj P.N., Blau W.J., Boland J.J., Coleman J.N. (2009). Silver Nanowire Networks as Flexible, Transparent, Conducting Films: Extremely High DC to Optical Conductivity Ratios. ACS Nano.

[12] Hu L., Kim H.S., Lee J.-Y., Peumans P., Cui Y. (2010). Scalable Coating and Properties of Transparent, Flexible, Silver Nanowire Electrodes. ACS Nano.

[13] Azuma K., Sakajiri K., Matsumoto H., Kang S., Watanabe J., Tokita M. (2014). Facile Fabrication of Transparent and Conductive Nanowire Networks by Wet Chemical Etching with an Electrospun Nanofiber Mask Template. Mater. Lett..

[14] Sepulveda-Mora S.B., Cloutier S.G. (2012). Figures of Merit for High-Performance Transparent Electrodes Using Dip-Coated Silver Nanowire Networks. J. Nanomater..

[15] Liu B.-T., Huang S.-X. (2014). Transparent Conductive Silver Nanowire Electrodes with High Resistance to Oxidation and Thermal Shock. RSC Adv..

[16] Ahn Y., Jeong Y., Lee Y. (2012). Improved Thermal Oxidation Stability of Solution-Processable Silver Nanowire Transparent Electrode by Reduced Graphene Oxide. ACS Appl. Mater. Interfaces.

[17] Im H.-G., Jang J., Jeon Y., Noh J., Jin J., Lee J.-Y., Bae B.-S. (2020). Flexible Transparent Crystalline-ITO/Ag Nanowire Hybrid Electrode with High Stability for Organic Optoelectronics. ACS Appl. Mater. Interfaces.

[18] Kim S., Kim S.Y., Kim J., Kim J.H. (2014). Highly Reliable AgNW/PEDOT: PSS Hybrid Films: Efficient Methods for Enhancing Transparency and Lowering Resistance and Haziness. J. Mater. Chem. C.

[19] Sun H., Wo Z., Su Y., Ma H., Zhang X. (2023). An Artificially Re-Structured PEDOT: PSS/konjac Glucomannan Sponge toward High-Performance Electromagnetic Interference Shielding from Gigahertz to Terahertz Bands. J. Mater. Chem. A.

[20] Chaudhary R.P., Das B., Oh S.I., Kim D.-S. (2019). Efficient Control of THz Transmission of PEDOT: PSS with Resonant Nano-Metamaterials. Sci. Rep..

[21] Liu G., Yang S., Lin H., Li Y., Lei J., Li Z.-M. (2023). PEDOT: PSS and AgNW Synergistically Contributed High Electromagnetic Shielding Performance for Polyurethane-Based Composite Coating. Compos. Part. A Appl. Sci. Manuf..

[22] Kim J., Maeng I., Jung J., Song H., Son J.-H., Kim K., Lee J., Kim C.-H., Chae G., Jun M. (2013). Terahertz Time-Domain Measurement of Non-Drude Conductivity in Silver Nanowire Thin Films for Transparent Electrode Applications. Appl. Phys. Lett..

[23] (2021). Standard Test Method for Haze and Luminous Transmittance of Transparent Plastics.

[24] Xue Q., Yao W., Liu J., Tian Q., Liu L., Li M., Lu Q., Peng R., Wu W. (2017). Facile Synthesis of Silver Nanowires with Different Aspect Ratios and Used as High-Performance Flexible Transparent Electrodes. Nanoscale Res. Lett..

[25] Graff A., Wagner D., Ditlbacher H., Kreibig U. (2005). Silver Nanowires. Eur. Phys. J. D.

[26] Li W., Xu X., Li W., Zhao Y., Chen M. (2018). Green Synthesis of Micron-Sized Silver Flakes and Their Application in Conductive Ink. J. Mater. Sci..

[27] Sun Y., Ren Y., Liu Y., Wen J., Okasinski J.S., Dean M.J. (2012). Ambient-stable tetragonal phase in silver nanostructures. Nat. Commun..

[28] Moskovits M. (2011). Preparation and Optical Properties of Silver Nanowires and Silver-Nanowire Thin Films. J. Colloid. Interface Sci..

[29] Gao Y., Jiang P., Liu D.F., Yuan H.J., Yan X.Q., Zhou Z.P., Wang J.X., Song L., Liu L.F., Zhou W.Y. (2003). Synthesis, Characterization and Self-Assembly of Silver Nanowires. Chem. Phys. Lett..

[30] Todd C.S., Chen X. (2019). Chemometric Evaluation of Ultraviolet-Visible (UV-Vis) Spectra for Characterization of Silver Nanowire Diameter and Yield. Appl. Spectrosc..

[31] Langford E.G., Shaughnessy K.D., Devore T.C., Lawrence D., Constantin C. (2016). Analysis of PEDOT: PSS Films after Sulfuric Acid Treatment on Silicon and Fused Silica Using FT-IR and UV-VIS. MRS Adv..

[32] Mao H., Feng J., Ma X., Wu C., Zhao X. (2012). One-Dimensional Silver Nanowires Synthesized by Self-Seeding Polyol Process. J. Nanopart. Res..

[33] Sahu N., Parija B., Panigrahi S. (2009). Fundamental Understanding and Modeling of Spin Coating Process: A Review. Indian J. Phys..

[34] Kong M., Garriga M., Reparaz J.S., Alonso M.I. (2022). Advanced Optical Characterization of PEDOT: PSS by Combining Spectroscopic Ellipsometry and Raman Scattering. ACS Omega.

[35] Toto E., Botti S., Laurenzi S., Gabriella Santonicola M. (2020). UV-induced modification of PEDOT: PSS-based nanocomposite films investigated by Raman microscopy mapping. Appl. Surf. Sci..

[36] Wang H., Zheng D., Zhang Y., Han L., Cao Z., Lu Z., Tan J. (2023). High-Performance Transparent Ultrabroadband Electromagnetic Radiation Shielding from Microwave toward Terahertz. ACS Appl. Mater. Interfaces.

[37] Xiong X., Zou C.-L., Ren X.-F., Liu A.-P., Ye Y.-X., Sun F.-W., Guo G.-C. (2013). Silver Nanowires for Photonics Applications. Laser Photonics Rev..

[38] Zozoulenko I., Singh A., Singh S.K., Gueskine V., Crispin X., Berggren M. (2018). Polarons, Bipolarons, and Absorption Spectroscopy of PEDOT. ACS Appl. Polym. Mater..

[39] Khanarian G., Joo J., Liu X.-Q., Eastman P., Werner D., O’Connell K., Trefonas P. (2013). The Optical and Electrical Properties of Silver Nanowire Mesh Films. J. Appl. Phys..

[40] Preston C., Xu Y., Han X., Munday J.N., Hu L. (2013). Optical Haze of Transparent and Conductive Silver Nanowire Films. Nano Res..

[41] Voronin A.S., Fadeev Y.V., Makeev M.O., Mikhalev P.A., Osipkov A.S., Provatorov A.S., Ryzhenko D.S., Yurkov G.Y., Simunin M.M., Karpova D.V. (2022). Low Cost Embedded Copper Mesh Based on Cracked Template for Highly Durability Transparent EMI Shielding Films. Materials.

[42] Pavlou C., Pastore Carbone M.G., Manikas A.C., Trakakis G., Koral C., Papari G., Andreone A., Galiotis C. (2021). Effective EMI Shielding Behaviour of Thin graphene/PMMA Nanolaminates in the THz Range. Nat. Commun..

[43] Przewłoka A., Smirnov S., Nefedova I., Krajewska A., Nefedov I.S., Demchenko P.S., Zykov D.V., Chebotarev V.S., But D.B., Stelmaszczyk K. (2021). Characterization of Silver Nanowire Layers in the Terahertz Frequency Range. Materials.

[44] Bril’ I., Voronin A., Fadeev Y., Pavlikov A., Govorun I., Podshivalov I., Parshin B., Makeev M., Mikhalev P., Afanasova K. (2024). Laser-Induced Silver Nanowires/Polymer Composites for Flexible Electronics and Electromagnetic Compatibility Application. Polymers.

[45] Macutkevic J., Seliuta D., Valusis G., Adomavicius R., Krotkus A., Kuzhir P., Paddubskaya A., Maksimenko S., Kuznetsov V., Mazov I. (2012). Multi-Walled Carbon. Nanotubes/PMMA Composites for THz Applications. Diam. Relat. Mater..

[46] Seo M.A., Lee J.W., Kim D.S. (2006). Dielectric Constant Engineering with Polymethylmethacrylate-Graphite Metastate Composites in the Terahertz Region. J. Appl. Phys..

[47] Macutkevic J., Adomavicius R., Krotkus A., Seliuta D., Valusis G., Maksimenko S., Kuzhir P., Batrakov K., Kuznetsov V., Moseenkov S. (2008). Terahertz Probing of Onion-Like Carbon-PMMA Composite Films. Diam. Relat. Mater..

[48] Huang Z., Chen H., Xu S., Chen L.Y., Huang Y., Ge Z., Ma W., Liang J., Fan F., Chang S. (2018). Graphene-Base Composites Combining Both Excellent Terahertz Shielding and Stealth Performance. Adv. Opt. Mater..

[49] Das A., Schutzius T.M., Megaridis C.M., Subhechha S., Wang T., Liu L. (2012). Quasi-Optical Terahertz Polarizers Enabled by Inkjet Printing of Carbon Nanocomposites. Appl. Phys. Lett..

[50] Agnandji E.N., Vigneras V., Miane J.L., Mounaix P. (2007). Shielding Effectiveness in Terahertz Domain of Monolayer-Doped Polyaniline Films. Electron. Lett..

[51] Venkatachalam S., Bertin D., Ducournau G., Lampin J.F., Hourlier D. (2016). Kapton-Derived Carbon as Efficient Terahertz Absorbers. Carbon.

[52] Voronin A.S., Fadeev Y.V., Ivanchenko F.S., Dobrosmyslov S.S., Makeev M.O., Mikhalev P.A., Osipkov A.S., Damaratsky L.A., Ryzhenko D.S., Yurkov G.Y. (2023). Original Concept of Cracked Template with Controlled Peeling of the Cells Perimeter for High Performance Transparent EMI Shielding Films. Surf. Interfaces.

[53] Jiang Z., Zhao S., Huang W., Chen L., Liu Y.-H. (2020). Embedded Flexible and Transparent Double-Layer Nickel-Mesh for High Shielding Efficiency. Opt. Express.

[54] Zhang Y., Dong H., Li Q., Mou N., Chen L., Zhang L. (2019). Double-Layer Metal Mesh Etched by Femtosecond Laser for High-Performance Electromagnetic Interference Shielding Window. RSC Adv..

[55] Chung S.-I., Kang T.-W., Kim P.K., Ha T.-G., Hong Y.-P. (2023). Highly Transparent Ka-/W-Band Electromagnetic Shielding Films Based on Double-Layered Metal Meshes. ACS Appl. Mater. Interfaces.

[56] Li H., Yin Z., Zhang C., Zhang Y., Deng R., Dong H., Wang S., Zhang L. (2022). Fabry–Perot Resonance-Suppressed Double-Layer Metal Mesh Window for Electromagnetic Interference Shielding. Opt. Lett..

[57] Wang H., Lu Z., Liu Y., Tan J., Ma L., Lin S. (2017). Double-Layer Interlaced Nested Multi-Ring Array Metallic Mesh for High-Performance Transparent Electromagnetic Interference Shielding. Opt. Lett..

[58] Gu J., Hu S., Ji H., Feng H., Zhao W., Wei J., Li M. (2020). Multi-Layer Silver Nanowire/polyethylene Terephthalate Mesh Structure for Highly Efficient Transparent Electromagnetic Interference Shielding. Nanotechnology.

[59] Lu Z., Ma L., Tan J., Wang H., Ding X. (2016). Transparent Multi-Layer Graphene/polyethylene Terephthalate Structures with Excellent Microwave Absorption and Electromagnetic Interference Shielding Performance. Nanoscale.

[60] Bennett H.E., Peck R.L., Burge D.K., Bennett J.M. (1969). Formation and Growth of Tarnish on Evaporated Silver Films. J. Appl. Phys..

[61] Elechiguerra J.L., Larios-Lopez L., Liu C., Garcia-Gutierrez D., Camacho-Bragado A., Yacaman M.J. (2005). Corrosion at the Nanoscale: The Case of Silver Nanowires and Nanoparticles. Chem. Mater..

[62] Kim S., Kim S.Y., Chung M.H., Kim J., Kim J.H. (2015). A One-Step Roll-to-Roll Process of Stable AgNW/PEDOT: PSS Solution Using Imidazole as a Mild Base for Highly Conductive and Transparent Films: Optimizations and Mechanisms. J. Mater. Chem. C.

