# Supplementary material for: THz Shielding Properties of Optically Transparent PEDOT:PSS/AgNW Composite Films and Their Sandwich Structures
Source: Polymers (Basel). 2025 Jan 24;17(3):321. doi: 10.3390/polym17030321 (PMC11821016; doi:10.3390/polym17030321)
Supplement: Supplementary file 1 [file polymers-17-00321-s001.zip › polymers-3372126-supplementary.pdf]

SUPPLEMENTARY MATERIALS

THZ SHIELDING PROPERTIES OF OPTICALLY TRANSPARENT PEDOT:PSS/AgNW THIN FILM COMPOSITE

by

Anton Voronin, Il'ya Bril, Mikhail Khodzitsky, Alexander Pavlikov, Mstislav Makeev, Pavel Mikhalev, Bogdan Parshin, Yuri Fadeev, Igor Tambasov, Sergey Nedelin, Nikita Zolotovsky, Mikhail Volochaev, Mikhail Simunin, Stanislav Khartov.

**P/ AgNW**			**P/ AgNW/ P**		
Number of Iteration	Average thickness, nm	Average roughness, nm	Number of Iteration	Average thickness, nm	Average roughness, nm
3	15	8	3	12	0.6
5	11	16	5	25	19
7	39	17	7	35	15
10	17	17	10	40	23
15	37	26	15	52	35

Table S1. Morphological characteristics of P/AgNW and P/ AgNW/ P structures.

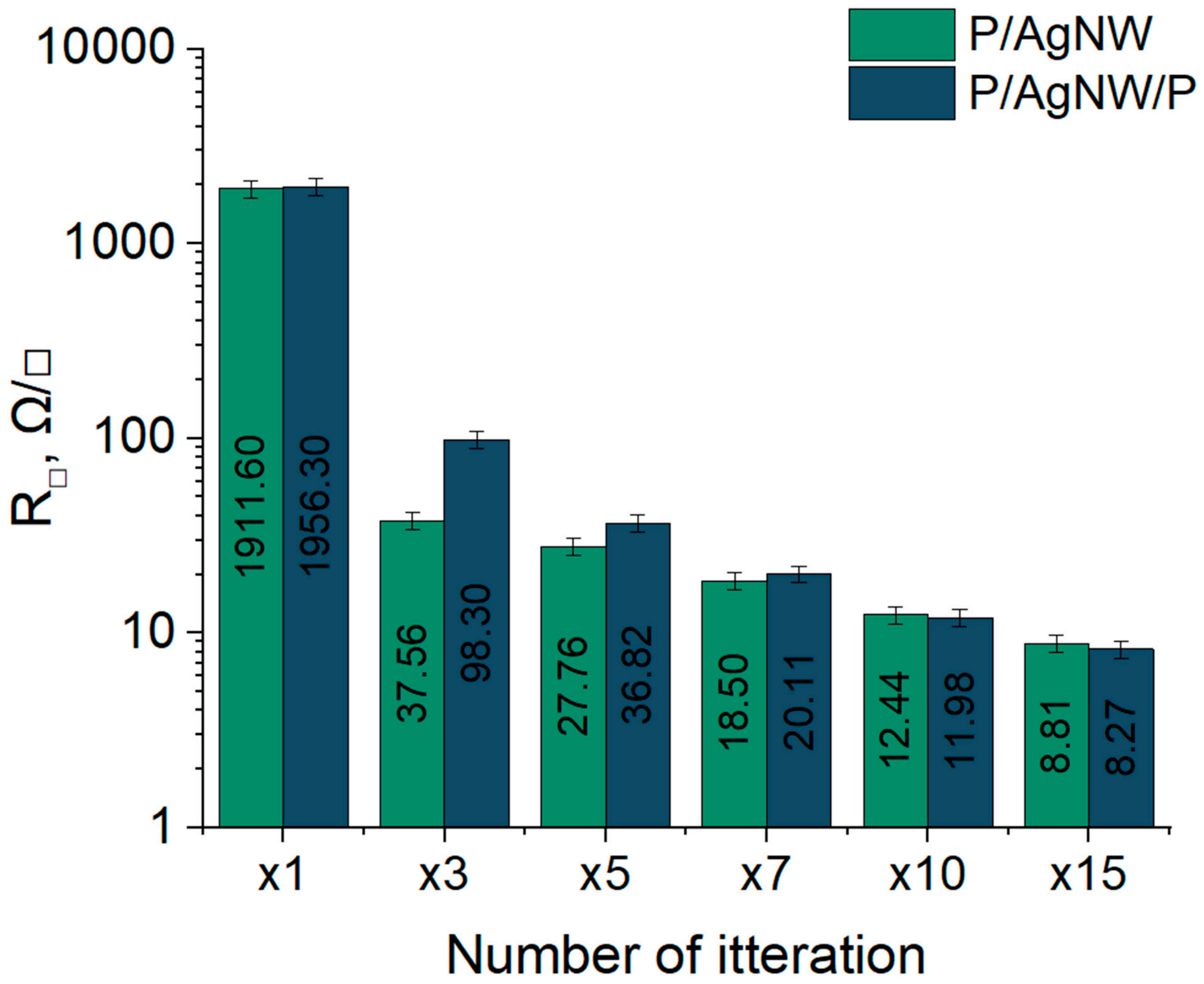

Figure S1. Sheet resistance of P/AgNW and P/AgNW/P composite films on PET substrate.

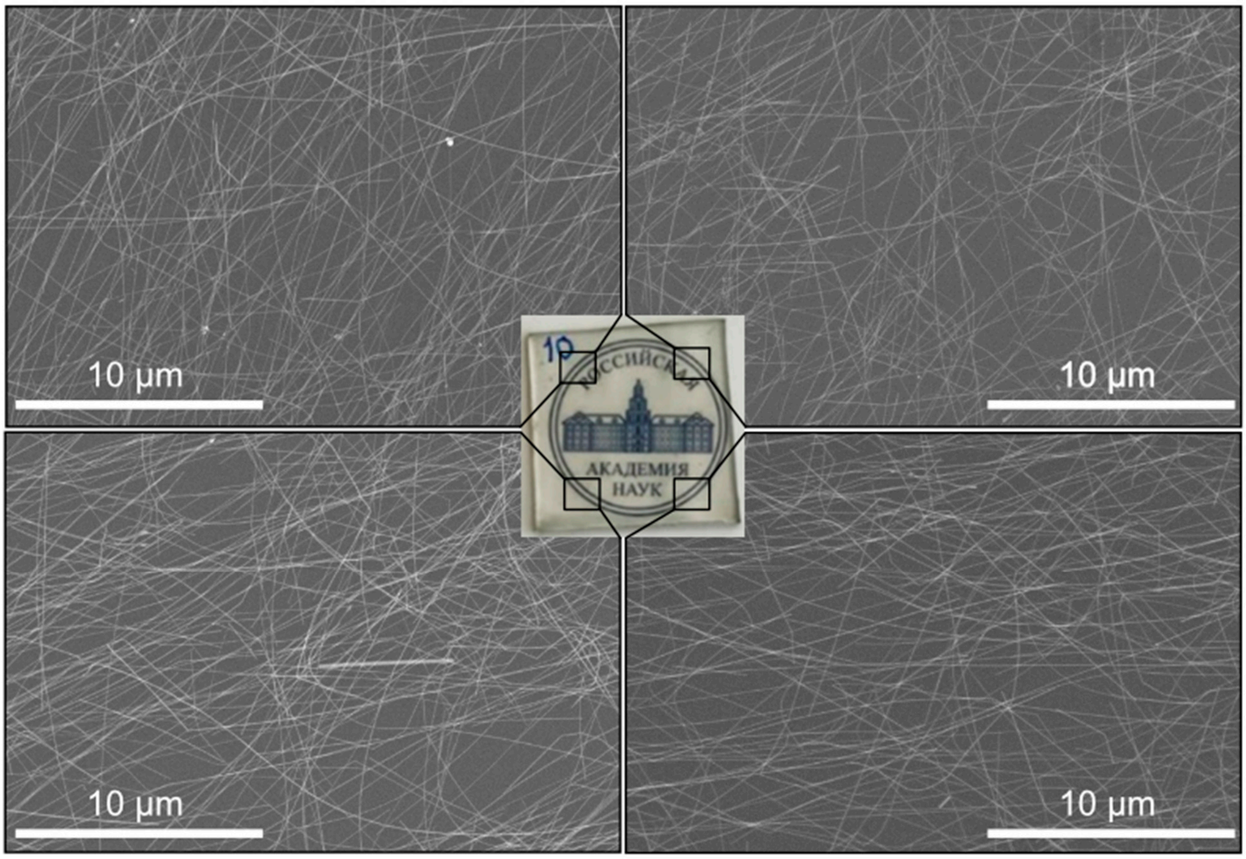

Figure S2. Homogenity of P/AgNW composite film in 10 itterations

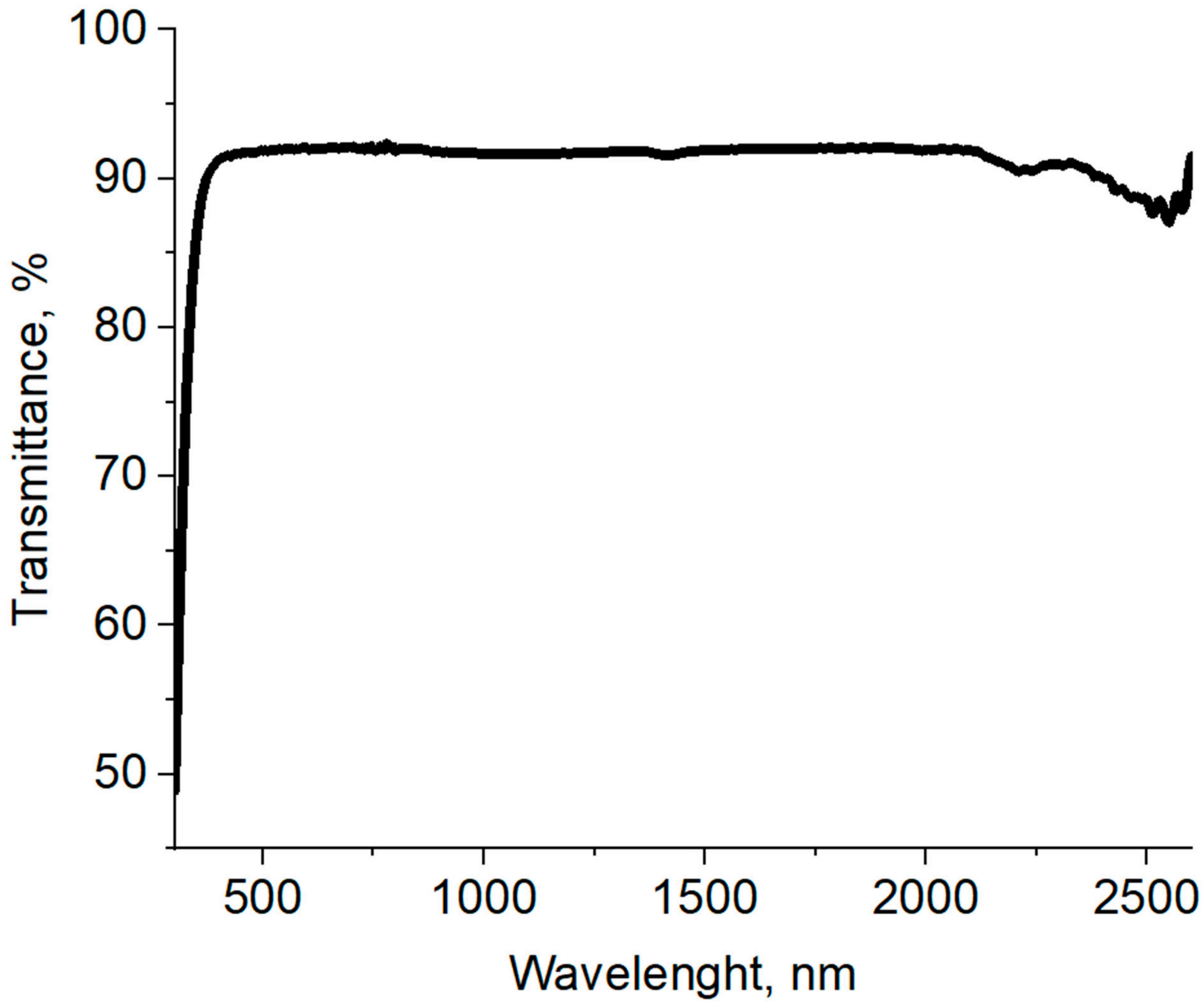

Figure S3. Glass transmittance spectra.

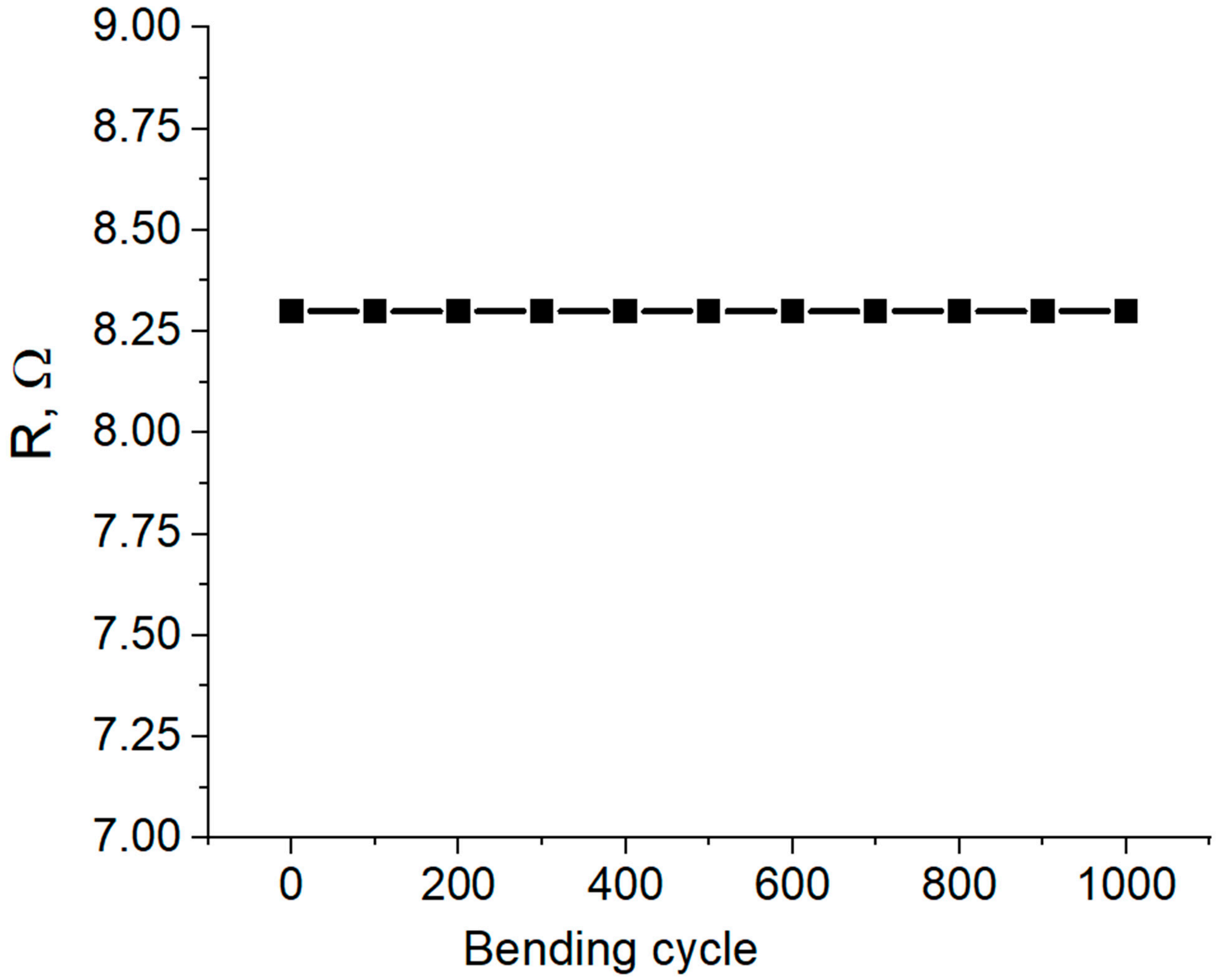

Figure S4. Stability to bending of P/AgNW (7 it.) structure.
